# Ligustrazine Prevents Intervertebral Disc Degeneration via Suppression of Aberrant TGF*β* Activation in Nucleus Pulposus Cells

**DOI:** 10.1155/2019/5601734

**Published:** 2019-12-02

**Authors:** Shufen Liu, Yuhao Cheng, Yuqi Tan, Jingcheng Dong, Qin Bian

**Affiliations:** ^1^Longhua Hospital, Shanghai University of Traditional Chinese Medicine, Shanghai, China; ^2^Institute for Cell Engineering, Johns Hopkins University School of Medicine, Baltimore, MD, USA; ^3^The Institutes of Integrative Medicine, Fudan University, Shanghai, China

## Abstract

**Objectives:**

Aberrant transforming growth factor *β* (TGF*β*) activation is detrimental to both nucleus pulposus (NP) cells and cartilage endplates (CEPs), which can lead to intervertebral disc degeneration (IDD). Ligustrazine (LIG) reduces the expression of inflammatory factors and TGF*β*1 in hypertrophic CEP to prevent IDD. In this study, we investigate the effects of LIG on NP cells and the TGF*β* signaling.

**Design:**

LIG was injected to the lumbar spinal instability (LSI) mouse model. The effect of LIG was evaluated by intervertebral disc (IVD) score in the LSI mouse model. The expression of activated TGF*β* was examined using immunostaining with pSmad2/3 antibody. The upright posture (UP) rat model was also treated and evaluated in the same manner to assess the effect of LIG. In *ex vivo* study, IVDs from four-week old mice were isolated and treated with 10^−5^, 10^−6^, and 10^−7^ M of LIG. We used western blot to detect activated TGF*β* expression. TGF*β*-treated human nucleus pulposus cells (HNPCs) were cotreated with optimized dose of LIG *in vitro*. Immunofluorescence staining was performed to determine pSmad2/3, connective tissue growth factor (CCN2), and aggrecan (ACAN) expression levels.

**Results:**

IVD score and the percentage of pSmad2/3+ NP cells were low in LIG-treated LSI mice in comparison with LSI mice, but close to the levels in the Sham group. Similarly, LIG reduced the overexpression of TGF*β*1 in NP cells. The inhibitory effect of LIG was dose dependent. A dose of 10^−5^ M LIG not only strongly attenuated Smad2/3 phosphorylation in TGF*β*-treated IVD *ex vivo* but also suppressed pSmad2/3, CCN2, and ACAN expression in TGF*β*-treated NP cells *in vitro*.

**Conclusions:**

LIG prevents IDD via suppression of TGF*β* overactivation in NP cells.

## 1. Introduction

Nucleus pulposus (NP) cells reside in the center of intervertebral disc (IVD) and play a key role in development of intervertebral disc degeneration (IDD) [1]. Mechanical loading triggers IDD by accelerating NP shrinkage and subsequently transits to fibroblast-like cells, which have decreased capacities in maintenance of IVD plasticity [2].

Ligustrazine (LIG) is an extract from Ligusticum wallichii Franchat (Chuanxiong), which is a traditional Chinese herb known for its wide usage in treating spinal degenerative diseases [3]. Two clinical trials (680 participants) found a compound that contains Chuanxiong, which relieved neck pain better in the short term than placebo in cervical IDD patients. One trail (60 participants) indicated an oral herbal formula with the component of Chuanxiong, which relieved pain better than Mobicox or Methycobal in treatment for cervical IDD [4]. An animal experiment revealed herb formula “Fufangqishe-Pill,” which prevents upright posture- (UP-) induced lumbar IDD in the rat model [5]. Further study showed the main effective component of “Fufangqishe-Pill,” LIG, decelerates the progression of lumbar IDD through inhibiting inflammatory factors such as IL-1*β*, COX-2, and iNOS in the upright posture (UP) rat model [6]. In addition to anti-inflammation effect, a standard traditional Chinese medicine library screening showed LIG exerts an antifibrosis effect via inhibition of TGF*β* [7]. Since IDD is also considered as a transition of fibrosis, we hypothesize that prevention of IDD by LIG is related to regulation of TGF*β* signaling.

Our previous work found the overexpression of TGF*β* accelerates cartilage endplate (CEP) hypertrophy and NP cell dysfunction, leading to IDD [2, 8]. Interestingly, LIG prevents CEP outgrowth and suppresses TGF*β*1 expression in hypertrophic CEP of IDD rats [9]. However, whether LIG has an antifibrotic effect on NP cells is not known. To investigate the effects of LIG on NP cells and explore its potential mechanism involving in TGF*β* signaling, we employed two IDD animal models to investigate the effects of LIG on NP cells and TGF*β* levels in different species and different mechanical loading patterns. We then isolated IVD from 4-week old mice and tested the dose response of LIG on TGF*β* signaling by western blot *ex vivo*. The optimized dose of LIG was further applied to NP cells *in vitro* to determine the TGF*β* signal downstream target gene expression of CCN2 and ACAN.

## 2. Materials and Methods

### 2.1. LIG Preparation

Ligustrazine phosphate (purity > 99%, MW: 252.21) was purchased from the Chinese Medicine and Biological Products Institute (Beijing, China). The solutions of ligustrazine phosphate were prepared in dimethylsulfoxide (DMSO, Sigma, USA) and diluted with culture media for the *in vitro* experiments. The final concentration of DMSO was no more than 0.1%.

### 2.2. Animal Models and LIG Administration

After intraperitoneal injection, anesthetized with ketamine (80 mg/kg) and xylazine (5 mg/kg), the 3rd–5th lumbar (L3–L5) spinous processes along with the supraspinous and interspinous ligaments of the 8-week old male C57BL/6J mice (28 g ± 5 g, Shanghai Laboratory Animal Center, China) were resected to induce the lumbar spine instability (LSI) mouse model. Sham operations were carried out only by detaching the posterior paravertebral muscles from the L3–L5 vertebrae. The operated mice were then intraperitoneally injected with ligustrazine hydrochloride (Nanning Maple Leaf Pharmaceutical Co., Ltd, China, 1 mL, 2 mg/d/per mouse) while the mice operated in the sham surgery were injected with the equal volume of saline solution at the same frequency and duration (once a day for one month). Protocol #PZSHUTCM190315024 was approved by the Shanghai University of Traditional Chinese Medicine Animal Care and Use Committee.

Sprague-Dawley (SD) (male, 1-month old, 200 g ± 20 g) rats (Shanghai Laboratory Animal Center, China) were anesthetized with 100 mg/kg ketamine by intraperitoneal injection. The forelimb surgery and customized cages force them to stand up which triggers IDD initiation as previously described [10]. Ligustrazine hydrochloride was administrated as described above (16 mL/kg·d, 4 mg/mL). Their lumbar spines were later dissected for further analysis. Protocol #PZSHUTCM190315024 was approved by the Shanghai University of Traditional Chinese Medicine Animal Care and Use Committee.

All animals were randomly divided into each group. All surgeries were performed in the laboratory, and drug administration was done in the animal facility. Animals were sacrificed before sample collection with CO_2_ exposure followed by cervical dislocation.

### 2.3. IVD *Ex Vivo* Model

The L1–L5 lumbar IVDs were removed under sterile conditions from 4-week-old C57BL/6J male mice (20 g ± 2 g, Shanghai Laboratory Animal Center, China). The collected IVDs were cultured in Dulbecco's Modified Eagle Medium (DMEM, Invitrogen, Carlsbad, CA, USA) supplemented with 1% penicillin-streptomycin (MediaTech, Dallas, TX, USA). The culture was then treated with 2 ng/mL of recombinant mouse TGF*β*1 with added LIG mixture at 0, 10^−7^ M, 10^−6^ M or 10^−5^ M dosage, respectively, overnight (rmTGF*β*1, 7666-MB-005, R&D, Minneapolis, MN, USA).

### 2.4. *In Vitro* Experiment

Human nucleus pulposus cells (HNPCs) were purchased from ScienCell research Laboratories (Catalog #4800) and were cultured with DMEM, 10% fetal bovine serum (FBS), and 1% penicillin-streptomycin. When the HNPC culture reached 70% confluency, they were starved by replacing 10% FBS DMEM culture medium with FBS-free culture medium for 24 h. Then, the HNPC cells were treated with 5 ng/mL rmTGF*β*1 combined with or without 10^−5^ M LIG or 0.01% DMSO (rmTGF*β*1 + Veh) for another 48 h.

### 2.5. Histochemistry, Immunohistochemistry, and Histomorphometry

The mice and rat specimens were fixed in 10% buffered formalin for 48 h, decalcified in 10% ethylenediaminetetraacetic acid (EDTA) (pH 7.4) for 14 days and 20% EDTA for 4 weeks, respectively, dehydrated, and embedded in paraffin. The mice specimens and the rat specimens of the L4-L5 spines were sectioned at 4 *μ*m and 7 *μ*m, respectively, and stained with Safranin O and fast green. Sections were incubated with primary antibodies to pSmad2/3 (1 : 200, sc-11769, Santa Cruz Biotechnology, Inc., Dallas, TX, USA), TGF*β*1 (1 : 100, Cell Signaling Technology Inc., MA, USA), ACAN (1 : 200, AB1031, MilliporeSigma, Billerica, MA, USA), CCN2 (1 : 400, ab6992, Abcam) at 4°C overnight. For immunohistochemical staining, a horseradish peroxidase-streptavidin detection system (Dako, Carpinteria, CA, USA) was subsequently used to detect the immunoactivity, followed by counterstaining with hematoxylin (Sigma-Aldrich). For the immunofluorescent assay, the slides were incubated with conjugated secondary antibodies conjugated at room temperature for 1 h while avoiding light and counterstained with 2-(4amidinophenyl)-6-indolecarbamidine dihydrochloride (DAPI). Morphometric study was performed by an image autoanalysis system (Olympus DP71).

### 2.6. Quantitative Histomorphometric Analysis

Quantitative histomorphometric analysis was conducted with Image-Pro Plus software version 6.0 (Media Cybernetics Inc., Rockville, MD, USA). IVD scores were obtained as described previously [11]. In detail, the organization of annulus fibrosus (AF), border between the AF and NP, cellularity of the NP, and matrix of the NP were assessed. The percentage of pSmad2/3-positive cells was obtained by counting the number of positive staining cells and the total number of cells in the NP region. The area of TGF*β*1-positive staining was calculated by measuring the positive staining area of region of interest that covers all cells in NP. The area of CCN2+ or ACAN+ per cell was measured by dividing the total positive staining area of CCN2 or ACAN+ by total cell numbers. Dividing the total positive staining area by total cell nucleus area, we get the percentage of pSmad2/3+ area per cell.

### 2.7. Western Blot

The cellular extraction from NP tissue in *ex vivo* assay was analyzed with a western blot. The cellular extraction was centrifuged, and the concentration of supernatants was evaluated by the DC protein assay (Bio-Rad Laboratories, Hercules, CA, USA), and then the proteins were separated by SDS-polyacrylamide gel electrophoresis and blotted on a polyvinylidene ﬂuoride membrane (Bio-Rad Laboratories). After incubation in antibodies, proteins were detected using an enhanced chemiluminescence kit (Amersham Biosciences, Pittsburgh, PA, USA). The target protein expression was examined with the following antibodies: mouse pSmad2 (1 : 1000, 3101, Cell Signaling Technology Inc., Danvers, MA, USA), Smad2 (1 : 1000, 3103, Cell Signaling Technology Inc.), and GAPDH (1 : 1 000, 8884, Cell Signaling Technology Inc.).

### 2.8. Statistical Analyses

The data are expressed as means ± standard error (SE), and statistical significance was calculated using one-way analysis of variance (ANOVA) followed by a post hoc LSD test (homogeneity of variance) and Tukey's test (heterogeneity of variance) using SPSS software (SPSS Inc., Chicago, USA). The significance level was defined as *p* < 0.05.

## 3. Results

### 3.1. LIG Maintains NP Cell Vacuoles and IVD Score in LSI Mice

**Figure 1:**
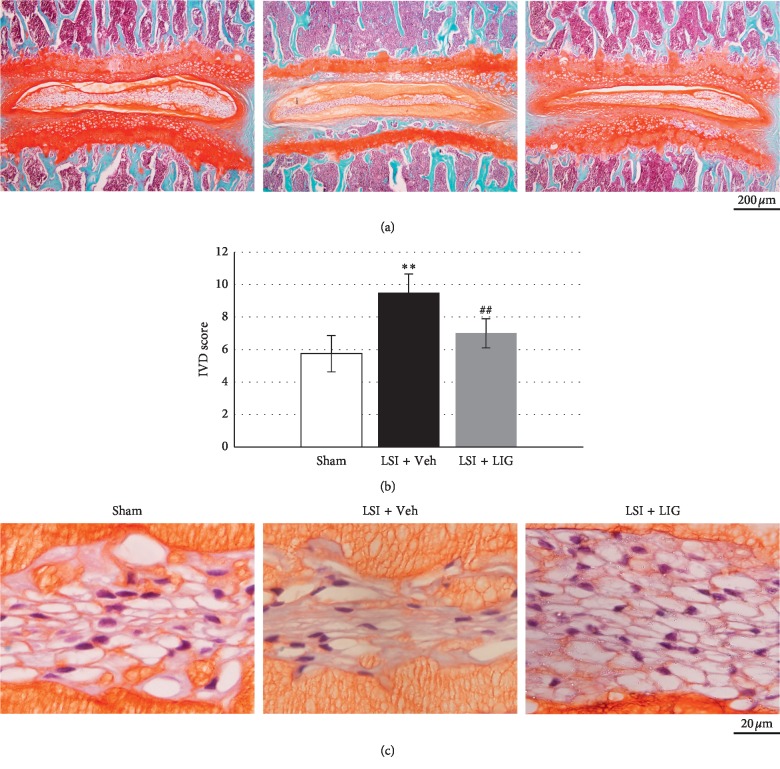
The effect of LIG on NP cell vacuoles and IVD score in LSI mice. (a) Representative images of the whole IVD sections in the LSI-induced mice model stained with Safranin O and fast green. (b) IVD score evaluated 4 weeks after LSI surgery. (c) Representative Safranin O staining images of the IVD sections showing the changes of NC cells in LSI + Veh, LSI + LIG, and sham-operated 8-week-old mice at 4 weeks after surgery. Each column represents the mean ± SE. *n* = 10 per group. ^*∗∗*^*p* < 0.01*vs* Sham, ^##^*p* < 0.01*vs* LSI + Veh.

The effect of LIG on NP cells *in vivo* was assessed by using the LSI mice model. LSI surgery induced significant IDD as shown by IVD score after 4 weeks. LIG treatment increased IVD score compared with vehicle (Veh) treatment (Figures 1(a) and 1(b)). Safranin O staining showed reduced vacuole sizes in NP cells after LSI surgery, whereas in the case where LSI mice were treated with LIG, the vacuole sizes were similar to those in Sham mice (Figure 1(c)). The maintenance of IVD score and NP cells' vacuole sizes indicated LIG preventing IDD progression.

### 3.2. LIG Decreases TGF*β* Activation in NP Cells of LSI Mice

**Figure 2:**
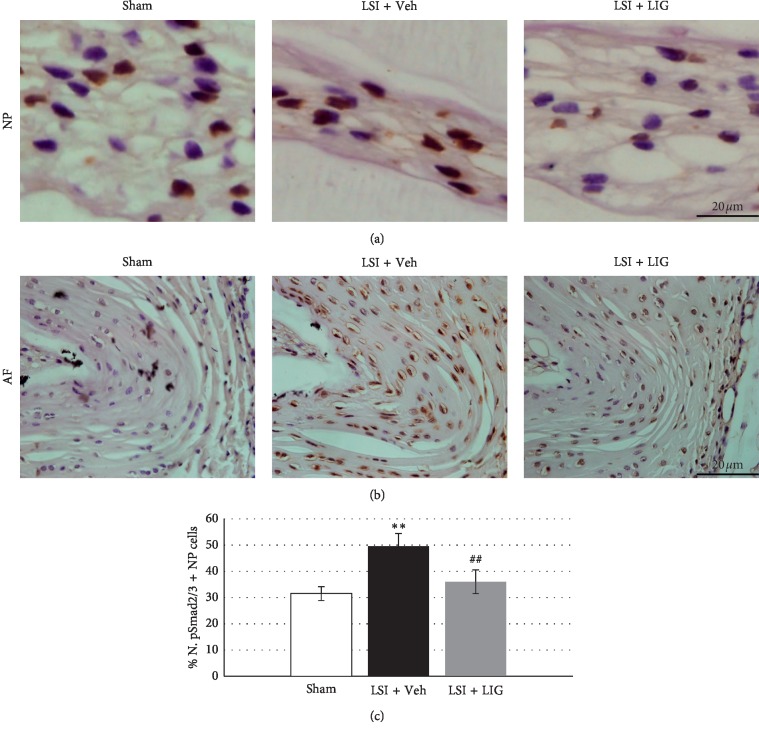
The effect of LIG on TGF*β* activation in NP cells of LSI mice. (a) Representative immunostaining images of IVD sections with antibody against pSmad2/3 (brown). Hematoxylin stains the nuclei purple. (b) Quantification of pSmad2/3+ cells in (c). Each column represents the mean ± SE. *n* = 8 per group. ^*∗∗*^*p* < 0.01*vs* Sham, ^##^*p* < 0.01*vs* LSI + Veh.

Vacuole sizes of NP cells are regulated by TGF*β* activation. To investigate the effect of LIG on TGF*β* activation during LSI-induced IDD, we examine the activation of TGF*β* with the immunochemical assay. It showed latent TGF*β* was activated after LSI surgery, indicated by significantly increased phosphorylated Smad2/3-positive (pSmad2/3+) cells in NP and annulus fibrosus (AF) (Figures 2(a)–2(c)). Decreased pSmad2/3+ cells were observed in the LIG treatment group relative to those in the Veh treatment group (Figures 2(a)–2(c)).

### 3.3. LIG Reduces TGF*β*1 Expression in NP Cells of UP Rats

**Figure 3:**
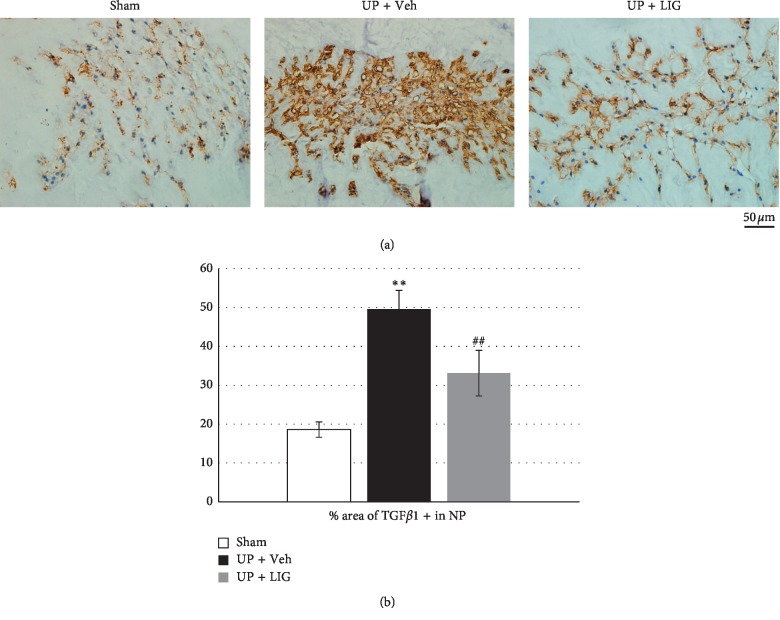
The effect of LIG on TGF*β*1 expression in NP of UP rats. (a) Representative immunostaining images of IVD sections with antibody against TGF*β*1 (brown). Hematoxylin stains the nuclei purple. (b) Quantification of TGF*β*1+ area *vs* NP area in (a). Each column represents the mean ± SE. *n* = 8 per group. ^*∗∗*^*p* < 0.01*vs* Sham, ^##^*p* < 0.01*vs* UP + Veh.

To test the effect of LIG on NP cells in different animal models, we employed a rat model in which IDD is induced by upright posture [10]. We found that TGF*β* expressions were significantly increased in NP cells of the UP + Veh group relative to the Sham group (Figures 3(a) and 3(b)). Continuous LIG treatment for 1 month significantly decreased TGF*β* expressions in NP cells relative to Veh treatment (Figures 3(a) and 3(b)).

### 3.4. The Effects of LIG on TGF*β* Activation in IVD Are Dose-Dependent

**Figure 4:**
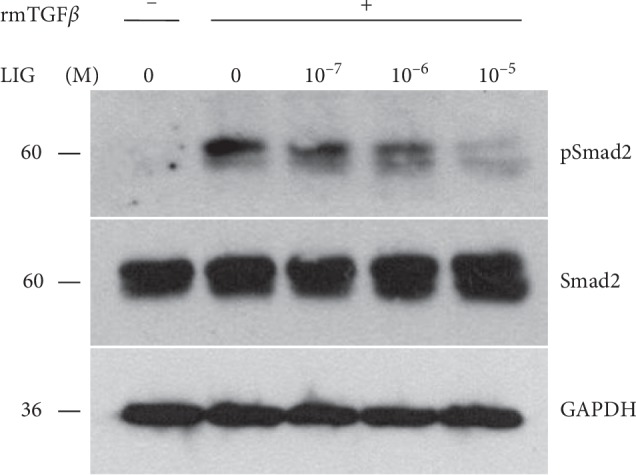
The dose-dependent effect of LIG on TGF*β* activation in mouse IVD *ex vivo*. Western blot analysis of pSmad2 and total Smad2 levels in the IVD, pretreated with 2 ng/mL of rmTGF*β*1 combined with 10^−5^, 10^−6^, and 10^−7^ M LIG.

To test whether the effects of LIG on TGF*β* activation are dose-dependent, we applied different doses of LIG on TGF*β* pretreated IVD *ex vivo*. After 24 h, the protein levels of pSmad2 were significantly increased by TGF*β* induction. The combination of LIG treatment decreased the levels of pSmad2 in a dose-dependent pattern. The dose of 10^−5^ M LIG exerted the most significant inhibitory effect on pSmad2 levels that were stimulated by exogenous TGF*β* (Figure 4). This dose is chosen for further *in vitro* experiment.

### 3.5. LIG Inhibits TGF*β* Downstream Factors CCN2 and ACAN in NP Cells

**Figure 5:**
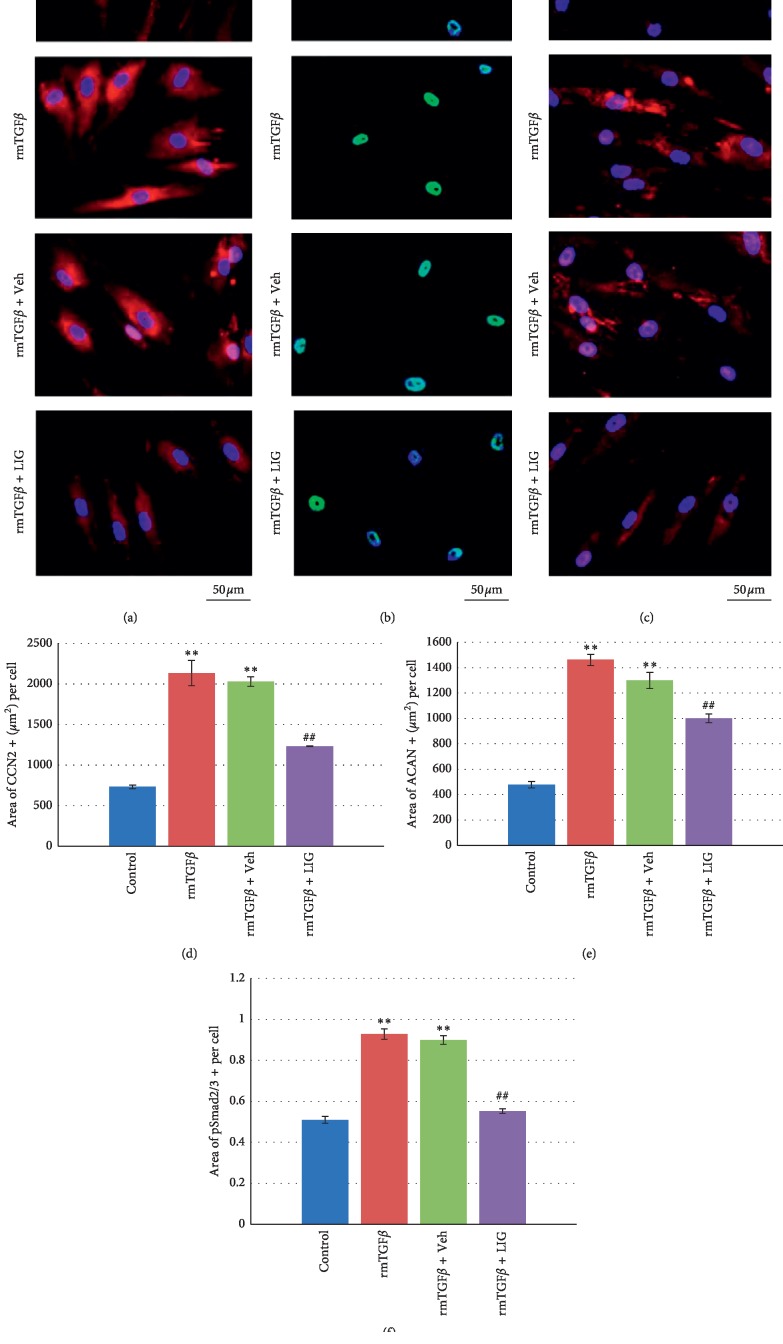
The effect of LIG on HNPC *in vitro*. (a–c) Representative immunostaining images of HNPC treated with 5 ng/mL rmTGF*β*1 combined with or without 10^−5^ M LIG, or 0.01% DMSO (rmTGF*β*1 + Veh) for CCN2 (a), pSmad2/3 (b), and ACAN (c). (d–f) Quantification of CCN2, pSmad2/3, and ACAN expressions in (a)–(c). Each column represents the mean ± SE of three independent experiments. ^*∗∗*^*p* < 0.01*vs* Control, ^##^*p* < 0.01*vs* rmTGF*β*.

We then investigate the effect of LIG on TGF*β* downstream factors *in vitro*. The levels of CCN2, which upregulates the synthesis of matrix proteins in IVDs [12–15], were significantly increased by exogenous TGF*β* induction (Figures 5(a) and 5(d)). The result is consistent with the increase of TGF*β* activity indicated by increased pSmad2/3+ cells (Figures 5(b) and 5(e)). Similarly, ACAN expression, upregulated by CCN2 [12, 16], was also increased in TGF*β*-stimulated NP cells (Figures 5(c) and 5(f)). As expected, the combination of 10^−5^ M of LIG treatment decreased the expressions of CCN2, ACAN, and pSmad2/3 in NP cells, indicating LIG inhibited TGF*β* activity (Figures 5(a)–5(f)).

## 4. Discussion

Nucleus pulposus cells, a remnant of embryonic notochord, are a unique cell type present in compartment of IVDs [17]. Overactivation of TGF*β* is a key molecular event that mediates NP cell transition, leading to IDD development. Recently, new therapies have been reported to prevent IDD via targeting NP cells and extracellular matrix remodeling such as microRNA-based therapy and melatonin [18, 19]. However, the therapy targeting NP cells through TGF*β* activity has not been reported.

Our study revealed that LIG prevented NP cell transition and maintained vacuole sizes of NP cells. LIG was reported to exert chondroprotective effect by inhibiting chondrocytes apoptosis [20], attenuating the interleukin 1*β* (IL-1*β*)-induced Glycosaminoglycan (GAG) degradation and matrix metalloproteinase-3 (MMP-3) expression, increasing cell viability through suppression of ROS production, maintaining mitochondrial membrane potential, and downregulating caspase-3 activity [21]. It is also a potent blocker of vasoconstriction and a strong scavenger of oxygen-free radicals. LIG enhances vascularization by increasing vessel volume, vessel surface, and vessel thickness, upregulating vascular endothelial growth factor (VEGF) in femoral heads [22]. However, LIG is barely able to increase vascularization on NP cells since NP cells are avascular.

We found the effect of LIG on NP cells is associated with inhibition of TGF*β* overactivation and TGF*β*1 expression, which is similar to several reports on other organs or cell types. For example, LIG decreases the expression of TGF*β*1 and reduces deposition of type III collagen during airway remodeling [23]. It also resists renal interstitial fibrosis by downregulation of TGF*β*1 and CCN2 and upregulation of Smad7 [24, 25]. LIG prevents and alleviates the development of liver fibrosis by downregulating TGF*β*1, T*β*RII, CCN2, and Smad2/3, and type I collagen while upregulating Smad7 [26, 27] and inhibits proliferation of hepatic stellate cells [28]. LIG prevents postoperative intra-abdominal adhesions by inhibition of pSmad2/3 expression and increasing the level of Smad7 in the peritoneal mesothelial cells [29]. Collectively, LIG prevents IDD via modulating TGF*β* signal.

There are also some limitations in our study. We observed the effect of LIG on animal models for one month which is relatively short. Long-term effect has not been assessed in this study. The potential downsides of long-term treatment may include antifibrotic effect on tissue repair such as scar tissue formation.

## 5. Conclusion

LIG prevents NP cell transition by suppression of TGF*β* overactivation. It can be developed as a therapeutic alternative for IDD.

## Data Availability

The data used to support the findings of this study are included within the article.
